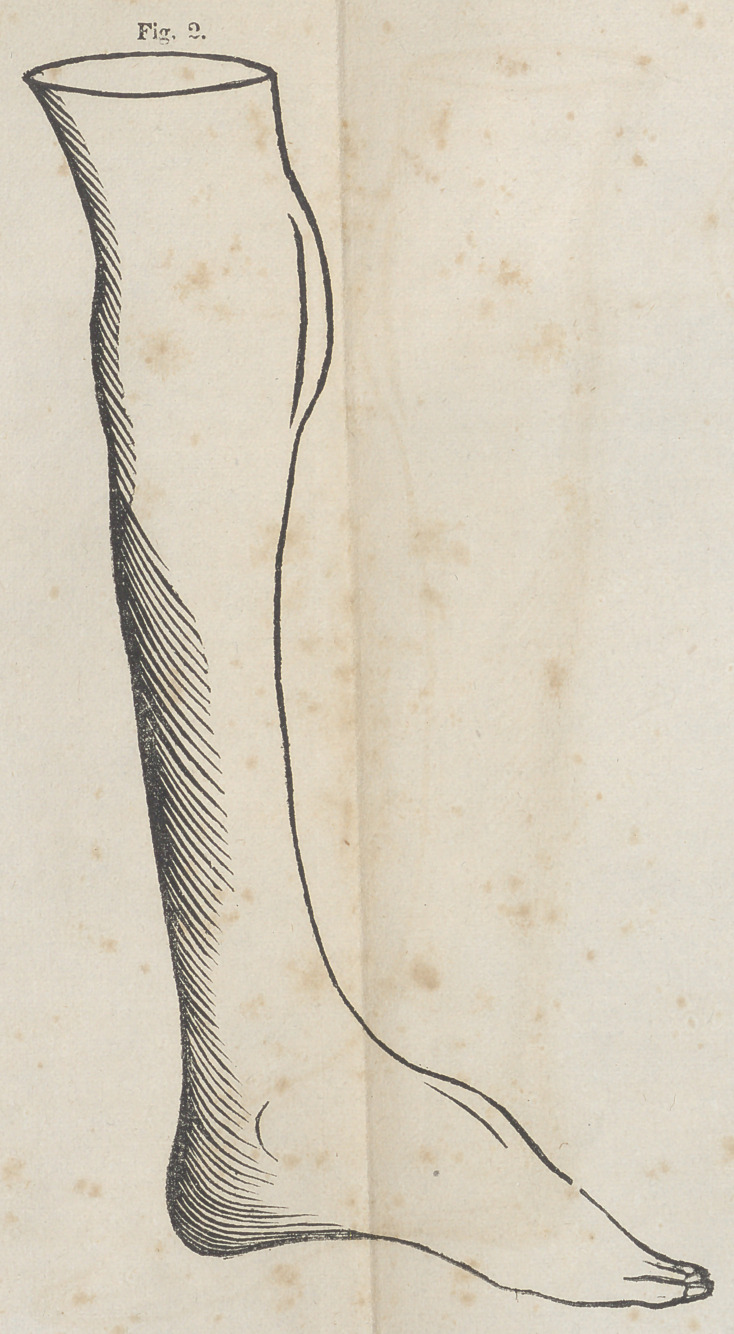# An Essay on Tenotomy; with Illustrative Cases

**Published:** 1840-10

**Authors:** S. B. Richardson

**Affiliations:** Lecturer on Anatomy and Surgery, &c., of Louisville, Ky.


### WESTERN JOURNAL

OF

### MEDICINE AND SURGERY.

---

OCTOBER, 1840.

---

### Art. I.—*An Essay on Tenotomy, with illustrative Cases.* By S. B. Richardson, M. D., Lecturer on Anatomy and Surgery, &c., of Louisville, Ky.

The operation of Tenotomy in the treatment of club-foot, which has met with such encouraging success recently in the hands of Stromeyer, of Hanover, and Bouvier and Duval, of Paris, (professed orthopedists of the continent of Europe) must be regarded by all who are familiar with the subject as a beautiful illustration of established physiological and pathological principles, and an undoubted advancement in the treatment of a very extensive but hitherto neglected class of individuals, found in every community.

In the *legitimate* employment of the term, it may be stated in the outset, that *tenotomy* must be regarded as a *discovery,* of such extensive application and importance, as to take rank with those great and acknowledged improvements in medical and surgical science, which have shed such brilliant lustre upon all that appertains to medicine, during the present age.

Like most true discoveries the merit of its origination cannot be awarded to the labors of a single individual, but it will be found upon a careful inquiry into its history, that the facts which led to its deduction were made known *long* anterior to *designed* tendinous section itself.

How encouraging should a fact like this be to those who are laboring in the field of observation, and thus become the benefactors of science, it is needless to insist. It rarely, in fact, falls to the lot of any *one* observer however qualified, to develope a single new principle in any department of knowledge. We are free to assert that every Newton will be found to have had a Gallileo, a Kepler, and other *observers* as his predecessors, and the remark will be found to apply not less appropriately to *medicine* than to *astronomy* and natural philosophy. Science, too, must be regarded, in some sense, as a *unit,* although artificially divided and sub-divided for convenience of study. It is not in the *nature* of true knowledge, but in the limited capacity of the human mind, connected with the comparatively *brief* period allowed for observation, that these artificial arrangements (in too many examples, dismemberments,) have been made and must be allowed to continue. What the God of nature has intimately associated should not in any case, cannot be dissevered except with the most injurious effects. Extra-professional scepticism among the learned as well as the uninformed is known to exist to some extent every where, in reference to the verity of medicine as *a science*—with how much truth, let those, who are immediately interested, give the answer, and abate the odium if found in any degree to exist. Some medical men, too, at no very remote date, and who have received the exalted appellation of *medical philosophers*, are known to have regarded the term *science* applied to medicine, as an unallowable assumption.

By these remarks, we do not wish to be understood as embracing or countenancing the sentiment just referred to; on the contrary we have too much attachment for, and confidence in practical medicine to detract one iota from her just merits, and the noble stand which she has recently assumed, and is destined to occupy (if rightly studied) as an *exact* science. It must in candor, however, be admitted, that medicine has not kept pace with those sciences par excellence entitled *exact*. This is doubtless referrable to more causes than one—chiefly however to the neglect of the inductive or Baconian (may it not be entitled Hippocratic ?) method of investigation, and the *multiplicity* of phenomena presented in *vital* manifestations, coupled with the heretofore almost inextinguishable thirst for theorizing, and *system building*, in medicine. The perception of this has led many distinguished physicians of the present age on the continent of Europe and elsewhere, to abandon the false route into which so many have strayed and wasted their energies, and to return to the path of patient observation,—admitting that there is not as yet a sufficient number of well established facts to serve as a basis for a theory, or, more properly, to evolve one or more controlling principles that are to organic what gravitation is to inorganic matter.

Without any farther preliminary remarks, we proceed to the subject which claims our immediate consideration.

When in the progress of pathological investigations it was ascertained, that *club-foot* essentially depends, on a *permanently shortened* or *contracted* state of certain muscles and tendons, connected with the movement of the foot upon the leg, and in some instances the toes upon the foot, the failure of every description of machinery to remedy it was easily accounted for. It was not less palpable, from what is observed in accidental rupture of the tendo achilles, as well as from direct experiment upon inferior animals, that nearly all obstacles to the *extreme* flexion of the foot upon the leg is overcome by a *section* of the tendons of the *extensor* muscles.

I have in my private cabinet a peculiarly interesting specimen of that variety of club-foot technically styled *Talipes valgus*, which I fortunately obtained for dissection whilst engaged in investigating this subject, in the capital of France. It is not apparent upon a careful inspection of it at the present time, that inflammatory irritation had ever invaded any of the elements or tissues which enter into the constitution of the foot and leg, nor was this the case when the preparation was made, now about two years ago—unless the thickening and induration of the skin and cellular substance, and the production of adventitious *bursæ mucosæ* corresponding with the *new points of pressure* upon the side or top of the foot, may be regarded as possessing this character.

The deformity, then, in the preparation before me, in which the points of the toes are thrown *outward* and backward, the inner margin of the foot downward, and the sole looking backward, is connected with a *permanently shortened* and rigid state of those tendons whose physiological action tends to incline the foot and toe in this particular position. Hence arises the principle or rule by which the deformity in *any of its species* or varieties may at once be recognized; viz: Those muscles with their corresponding tendons, whose individual or combined action, when *strongly contracted, tend* to its production in a *normal* condition of the parts, are concerned in producing or maintaining the deformity in question. Deformities of the foot (which may be congenital or acquired) have the generic appellation of *Talipes* referring to the ankle, or pastern of a beast, or more classically, perhaps, *Kyllopodia.** Of this, three varieties are distinguishable. 1st. That in which it is turned inward, (*vara,*) 2d. That in which the foot is turned outward, (*valga.;*) and 3d. That in which the foot, from the *extreme extension*, is turned upon itself, and rests upon the ground by the *superior surface* of its phalangeal extremity, (*equina*) called, also, phalangeal club-foot.

It was suggested long ago, that these deformities were caused *primarily* by an irregular conformation of one or more of the tarsal bones, by a defect of *equilibrium* in the action of the muscles which move the foot, or an abnormal insertion of one or more of their tendons. To these I will take the liberty of adding an ingenious supposition of M. Duval, of Paris, which I received from him in a conversation upon this subject. This gentleman is well known as a practical orthopedist of distinction in that capital, and by his numerous memoirs delivered before the French Institute, and the Royal Academy of medicine, upon subjects connected with his speciality. He holds that the *primary* cause of these deformities resides in the cerebro-spinal axis, and that they are in

* From *kullos*, crooked, and *pous-podos*, foot.

fact to be referred to the nervous system *originally*, and secondarily to spasmodic contraction of certain muscles.

This is confessedly hypothetical, especially in reference to much the largest class of these affections which are congenital—but whilst little is, or perhaps ever will be certainly known, of the pathology of the fœtus in utero, we confess that there seems to be some ground for the supposition when we consider the violent and protracted motions of the fœtus in utero, sometimes observed, which, coupled with some other facts connected with practical obstetricy, justifies the inference that the fœtus in utero is more subject to *nervous and spasmodic affections,* than to any other class of diseases.

We are inclined to believe, moreover, that an unnatural and *distorted posture* of the fœtus, during the early periods of gestation, may have occasional agency in the production and maintenance of these deformed developments.

It might be considered appropriate here to consider the supposed influences of mental impression or emotion of the mother during gestation, as an original *primordial* cause of *these,* with other deformities and *marks.* But, whilst this is a deep rooted impression in the popular mind, possessing like most kindred prejudices connected with *occult* matters of science, some *apparent* facts upon which they are predicated; it must be acknowledged that it has been a fertile subject of speculation with physiological and obstetric writers, from which nothing valuable in science has ever resulted. The facts we know, and these are sufficient for our purpose.

Not so with those influences connected with *hereditary descent*—these are ever operative, and extensively so. Like begets like in disease, as well as in health. The records of the profession abound with illustrations of the fact in reference to the deformities under consideration, and those which are congenital must continue to multiply annually in increasing numbers, unless more attention is bestowed upon the subject of their nature, and the improved methods which science is holding out for their successful treatment.

I could cite numerous instances which have come to my knowledge, in which club-footed children were the offspring of a parent with like deformity; I will cite however only one, the most remarkable. I was consulted in the autumn of 1839 by a young gentleman of the South with double club foot, who assured me that an instance presented in Mississippi in which an entire family of six or seven children, male and female, were the offspring of a club footed father!

Nevertheless it must be regretted, with all the facts and arguments before us, that in this, as well as in many other morbid conditions appertaining to the pathology of *extra* uterine life, the original or starting point of diseased action is surrounded with such obscurity, that its correct interpretation will not speedily, if ever, be obtained.

Whatever may be the cause of any of the varieties of the deformity under consideration—whether the case be congenital or accidental, the weight of the body in the erect position and during locomotion powerfully contributes to augment it. This is especially the case during adolescence, where the subject has been accustomed to an *active life*, and the trunk and superior extremities are correspondingly developed, or where obesity obtains either at the period referred to, or in mature life. I have been repeatedly consulted by those who upon inquiry assured me, that their deformities had augmented more in a few months about the period of life or under the circumstances referred to than in as many years at any former time.

In the first and second species, in which the torsion of the foot is inwards or outwards, the os calcis, cuboides, scaphoides and cuneiform bones experience an abnormal rotation upon the antero-posterior axis of the foot. In the first species, in which the torsion is inwards and which is by far the most common, the os calcis is thrown outwards with its posterior extremity into which the tendo-achilles is inserted, more or less drawn upward, the external margin, and sometimes in extreme *torsion*, part of the *superior surface* of the os cuboides, presents downwards, and the internal tuberosity of the scaphoides is found beneath the internal maleolus. In addition, the points of the toes with the foot are usually thrown transversely inwards, or inwards and backwards and upwards, the plantar surface backwards and upwards, its inner margin presents upwards, and sometimes *forwards and upwards*, and as already indicated the outer margin downwards or downwards and backwards. The muscles with their corresponding tendons ordinarily found *contracted* and concerned in maintaining this deformity, are, the gastrocnemius, soleus and plantaris *mainly*, sometimes the tibialis posticus and anticus are involved, the latter becoming in some degree an *extensor*, and *adductor* of the foot. The flexor propius pollicis, flexors brevae, longus and communis together with the fascia plantaris may or may not be seriously implicated. The peronæi, as might be supposed, are unduly stretched and elongated.

The second species, (K. valga.) of which the specimen above spoken of is an *extreme* example, is much the least common of the three varieties and is *rarely* met with. When seen the posture of the foot will be found, as described already, with the points of the toes directed outwards, outwards and backwards, or backwards and downwards, the inner margin of the foot downwards, or downwards and forwards, the outer margin directed backward or backward and upward—the calcaneum sometimes not at all, generally, we believe, *less* elevated than in the first species. The ossa cuneiformi and metatarsal range experience the same description of rotation as in the first species, but in an opposite direction; the plantar face of the foot, which presents backward and outward, is very *concave* and filled with deep depressions or sulci—the dorsal face is correspondingly *convex*, the internal margin is apparently shortened and offers a considerable concavity, whilst its external border is correspondingly convex and elongated—the great toe is *peculiarly* projecting, and drawn upward and outward, at the same time that the remaining toes are often turned in an opposite direction.

The principal muscles constituting the calf the leg are not as much contracted in this as in the first species described; whilst the *peronæi*, tibialis posticus, flexor propius pollicis, flexor brevis and longus are found extremely tense, contracted, and shortened. The flexors appropriated to the small toe and the one next to it, in the specimen alluded to, are so powerfully contracted as to have *articulating surfaces* at the metatarso-phalangeal articulations, and draw the points of these toes directly toward the external maleolus, whereby their phalanges are reversed, and applied upon their corresponding metatarsal bones. The *elongated* tendons in this deformity are, the tibialis anticus, extensor proprius, and extensor communis digitorum pedis.

I have not had occasion to remark the existence of *anchylosed* club foot—nevertheless we are informed by pathologists that in very ancient cases, the bones lose their natural form, and even become partially or completely *anchylosed*.

In the third species (equina) the heel is forcibly drawn up, and maintained *permanently* in that position by the contracted condition of the gastrocnemius, soleus &c.; the dorsum of the foot forms a very *open* angle with the leg, its plantar face very concave, the weight of the body is received upon the heads of the metatarsal bones, which form a right angle with the toes.

It only remains to be stated on this branch of our subject, that nature, ever true to herself, establishes in every species and variety of Kyllopodia in which the *individual has walked*, such changes in the epidermis, skin, and sub-cutaneous cellular tissue corresponding with those surfaces which receive the weight of the body in progression, as in effect to constitute a new plantar surface or sole of the foot, whilst the proper plantar surface possesses much of the softness, pliancy, and delicacy of structure found in the cutaneous covering of the dorsum of the foot in its normal position.

The treatment of Kyllopodia, before as well as since its pathology was in some degree rightly estimated, until very recently was attempted by machinery alone. Whilst these measures have been attended with only very partial success, even after the most protracted and *painful* employment of them,—the fact must not be concealed that occasional cures have followed their use in early periods of life, before the muscles and tendons had acquired their adult development and rigidity. Most cases, however, have been either abandoned altogether, or at most the *club-foot boot* has been applied, the ordinary effect of which has been only to harass and irritate the patient.

These unsatisfactory results led surgeons to conceive the division of the tendons as a means of *facilitating* the correction of these deformities,—and although this operation was first performed upon the living human subject by Loreny, as early as 1784, yet we feel confident that the facts which led him to its *deduction and execution* were known and *recorded* as early at lest as 1769.

The following extracts and translations, from the compilations of M. Paul will place this latter fact beyond the reach of cavil or doubt.

In discussing the subject of wounds of the Tendo Achilles, the writer says:—

"The opinions of the most distinguished authors on Surgery are very much divided, respecting the course to be pursued, when the tendo achilles has been divided by a cutting instrument, or otherwise. Some of these, among whom is M. de la Faye content themselves with an *approximation* of the ends of the divided tendon, and thus maintaining them by posture and the bandage, *without suture*—the majority however advise the latter. M. Heister thinks with de la F. that posture and the bandage, should be employed when these are sufficient to maintain the extremities of the divided tendon in *contact*, but that the *suture* is sometimes *indispensable* for this purpose. *

"M. Mollinelli has given us in the history of the Academy of Bologne (Commentarie Acadamie Scientiari Bononiensi Tom. 2 Par. 1 pp. 189, 196) the details of some cases which throw much light upon this matter, and perhaps settle the question." †

The cases are four in number, in all of which the tendo achilles had been partially or completely divided by accident; in all of them, M. M. removed *sections* of greater or less extent of the extremities of the *gangrened* tendons, in all, the lost substance of the tendon was *re-produced*, and the function of the extremity restored, in periods varying from

* Memoirs, 28th Article, intended to serve as a history of Surgery in the 18th century, and as a suppliment, to 3d volume of the Institutes of Surgery of M. Laurent Heister. By Dr. Paul, 1773. L. Histoire de l'Acadamie de Bologne Tom. 2. Par. 1.

† See Institutions de Chirurgie Par. 2. Sec. 6, Chap. on Suture of the Tendons.

six weeks to three months. These casese are interesting in several points of view, as they prove conclusively the re-production of lost tendon, and, as they are reported in a very succinct manner, we shall extract them without abridgement.

"*Case* 1. A man, aged 48 years, of bad constitution, living in a marshy district, received a wound which divided the tendo achilles of one leg in *nearly half of its thickness.* Some days elapsed before he was brought to the hospital, when the leg was found already tumid, particularly the calf. The inferior margin of the wound had assumed a livid green color, and upon pressure of it a similar colored sanies issued from every part. By the stilet M. Mollinelli discovered a sinus of rather large size, extending nearly to the middle of the calf of the leg, and found a portion of the tendon separated from the integuments inferiorly and laterally. For the purpose of exposing that part of the tendon, the wound was dilated in every direction, when the tendon was found larger and harder than natural, and unfortunately it was found to be gangrenous to the extent of two fingers breadth in length. After vainly attempting to restore the gangrened portion, there remained no other alternative but its extirpation with the knife—the operation for which was dauntlessly sustained by the patient. During this operation the thickened margins of the wound were likewise removed. Soothing and anodyne applications were made to the wound, care being taken not to tighten the bandages beyond what was essential to maintain the dressings, the patient never suffering unless they were tightened beyond this necessity. Succeeding to this, venesection was had recourse to several times, when the leg and wound began to assume a better appearance and condition, nevertheless some uneasiness was constantly experienced in the lower extremity of the divided tendon, which continued tumid and elevated slightly above the adjacent integuments and, to increase the evil, became *gangrenous.* All the remedies had recourse to, proving abortive, there remained no other alternative than the iron or the knife. The proximity of the heel, being not more than a finger's breadth distant, precluded the first, and M. M. not wishing to abandon the patient to his apparently hopeless condition, boldly removed the gangrened portion the *second time.* The same treatment as before was continued, the most perfect rest of the limb being enjoined. The wound was besides dressed with suppuratives. By this course of treatment, the tumefaction gradually abated, and in two and a half months the wound was cicatrized. The space left by the loss of tendon was filled with a fungous growth, which was manifest after the cicatrization was complete by an elevation at the original seat of injury. The foot having been kept for a long period in *extension*, it was apprehended that this position would continue after cicatrization of the wound, and thereby entail lameness upon the patient for life. The result, however, was more favorable than anticipated, for although some difficulty was experienced in applying the heel to the ground when the patient began to walk, this inconvenience very speedily ceased and finally *he walked with the same facility as before the accident.*

"*Case* 2, Was that of a young man, 24 years of age, who divided his tendo achilles with a scythe to the extent of about one third of its thickness. Pain with violent fever succeeded, when M. M. did not hesitate to make the partial tendinous section *complete*, and opened a sinus besides, which had formed beneath the integuments. The superior extremity withdrew to a small extent upon division; the dressings employed were light and in a short time recovery was *complete.*

"*Case* 3, Is that of a man aged 32 years, who received a very severe wound of the achilles tendon, almost completely severing it transversely. The tendon of the plantaris muscle was so much relaxed as to project from the wound—the projecting tendon was *removed*, and the margins of the wound opened. Extensive abscesses formed beneath the integuments extending to the malleoli which were opened. By the use of retentive bandages, in about two and a half months, this individual recovered the *perfect use* of the injured extremity, and no other effect of the accident remained but an elevation or knot over the site of injury.

"The 4th and last case of M. Mollinelli, was that of a young man of uncommon size, and muscular development, who completely divided his tendo achilles about two fingers breadth from the heel. The accident was disregarded for several days. The superior extremity of the divided tendon had retracted beneath the integuments—the lower extremity was hard and tumefied, especially its wounded surface. Efforts were made to overcome the necessity of using the knife, but these were thwarted by the obstinacy of the disease. Tumefaction and induration daily increased, sinuses formed, some of which broke, hemorrhage ensued from the wound. In this extremity M. M. opened the sinuses over the tendon, and amputated its tumefied and indurated extremity. The disease persisting, the inferior end of the tendon began to swell—this did not attain the size of the first, but its persistence required its amputation, when not more than two lines of tendon remained attached to the calcis. Succeeding the last operation a new aspect presented, and in a short time the wound closed by granulations. The patient would permit no other dressings than a simple retentive bandage, nor would he allow flexion or extension of the foot. From his obstinacy, the os calcis was found considerably drawn upward by the time he commenced walking, so as to prevent the foot *being placed flat upon the floor*—nevertheless, the recovery was so perfect that it was with difficulty that any difference in the functions of the two members could be perceived—the injured member admitting of every variety of motion."

The commentator, M. Paul, makes the following just reflections. "We perceive by the observations of M. Mollinelli, as well as by a single case of Gaingeot, that it is not necessary in order to insure union of the divided tendo achilles, to *approximate the extremities,* either by posture and the bandage, or by the suture, as had been previously practiced. Besides, in the observations presented, the *loss of substance was such* that no means could possibly effect it.

"These important observations had not been cited by any preceding writer on surgery, and it is surprising that they should be unknown to so learned an author as M. Heister."

In the January number of the Journal of Medicine of Dijon, for 1769, (pp. 56, 78,) *M. Hoin,* a member of the Academy of Medicine and Surgery of that city, wherein he resided, reported a number of experiments confirmatory of the observations of M. Mollinelli. He divided the tendo achilles of cats and dogs, partially and completely, and although *abandoned without special treatment,* they invariably recovered. The exclusion of atmospheric air from wounds was regarded as indispensable to safety and success, by many of the older surgeons, but this precaution as stated was not observed in the above experiments.

These observations and experiments undoubtedly led to the suggestion of Tenotomy, or the division of tendons, as a surgical operation, which, as far as the records of the art inform us, was first performed by *Lorenz*, of Frankfort, who has been already spoken of. He practiced a section of the tendo achilles in the case of a young woman, aged 17 years, the 26th March, 1794, by making a *complete division of all the soft parts* embracing the tendon, from its anterior limits posteriorly. This allowed of an immediate descent of the os calcis, to the extent of *two inches.* A bandage maintained it in that position, and in six weeks the wound was closed. This observation was published by Thelenius in 1789.

In the year 1811, Dr. Michaelis, of *Marbourg*, divided the tendons in three cases of K. equina and one of K. vara—in the latter the tendon of the *tibialis anticus* was *alone* divided. He assures us that the first three cases were restored in a month.

Sartorius, of the Duchy of Nassau, in 1812 proposed and executed another method of operating. He made a longitudinal incision along the side, and parallel with the tendo achilles, opened its theca to the extent of admitting a grooved director, upon which a section of the tendon was made with a straight bistoury.

The individual, he assures us, upon whom this operation was performed walked upon the back or top of the foot;—the foot was flexed immediately after the operation and by maintaining it thus the cure was nearly radical.

*Delpech* was the first to propose a division of the tendon *without dividing the skin* covering it. The 9th May, 1816, he operated upon a child 6 years of age, by transfixing the leg in front of the tendon with a sharp pointed bistoury, incising the skin to the extent of an inch on each side, and then dividing the tendon with a *convex* knife, without further division of the integuments. Delpech favored *union of the divided* tendon, by maintaining the foot in *extension for twenty-eight days*, when he supposed this result had taken place. Then *gradual* flexion of the foot upon the leg was effected, with a view of *elongating* the newly formed medium, uniting the cut extremities of the tendon. The flexion was painful, but in 8 days the foot was brought to its normal position. This operation was followed by high *inflammatory irritation*, and the wound at the expiration of three months had *not closed*, whilst some obliquity of the foot existed. Twenty years after this operation, the subject of it who was (and probably is yet,) living, was examined by *M. Bouviel*, of Paris, and reported to the Royal Academy of Medicine. He mentions the gratifying fact that there remained at the time his examination was made, only a slight deviation or lameness, not sufficient to prevent the individual from making long walks free from fatigue.

This partial success of Delpech did not encourage him to operate again, nor had he any imitators until 1833 '4, when *Stromeyer*, of Hanover, published six new cases of tenotomy after the *manner* of Delpech modified.

This modification consisted in passing a convex, sharp pointed knife in *front* of the tendon, (thereby endangering, we believe, a wound of the posterior tibial vessels and nerves) allowing the point barely to issue through the integuments on the opposite side—when the operation was completed without enlarging the cutaneous punctures.

He flexed the foot at the expiration of ten days in adult cases, and five for children,—in this particular at least, imitating the practice of Delpech.

The operation and treatment *failed* in the case of a child, in which eight days were allowed to elapse after the operation, before flexion &c. was commenced.

Five out of the six cases were cured, four of which were K. vara and two K. equina. The youngest was aged seven years, the eldest 32. Mechanical means alone had been used unsuccessfully in two of these cases: third case apparently cured by the same treatment, suffered a *relapse.* In one case the operation of tenotomy was preceded by the use of mechanical means, in the other five tenotomy was first performed.

In one case section of the tendo achilles was preceded by a section of the tendon of the great toe—in another it was succeeded by a similar operation upon that of the extensor proprius pollicis pedis.

The duration of treatment was a month in the two first cases, five weeks for the third, six weeks for the fourth, and two months for the fifth case.

We are indebted for most of the facts in the brief history of the different manuals of tenotomy, which had been practiced prior to the year 1835, detailed in the last few pages, to the report of M. M. Blandin, Emery, and Velpeau, made to the Royal Academy of Medicine of Paris, upon "a memoir on the treatment of Club-foot by a section of the tendo achilles. By M. Bouvier."

From this report, a copy of which was presented to me by M. Bouvier, (through whose courtesy I had the opportunity of visiting his private orthopedic establishment,) I will in further illustration of this, and some other matters appertaining to this branch of our subject, add a few extracts and translations.

"Shortly after the publication of Stromeyer's cases, and in the month of February, 1835, M. Bouvier, assisted by the younger Berard, intended operating for club-foot, but for the unfortunate death of the patient from acute disease. Toward the conclusion of that year Bouvier and Duval performed tenotomy almost simultaneously in Paris."

As it is believed that Bouvier (whose manual for tenotomy we witnessed as practiced by himself at the Hotel Dieu of Paris,) has made some important and practical modifications in this particular, we will first, for the benefit of those who may determine to execute the operation, make the following literal translation.

"Bouvier has suppressed one of the openings and never makes but *one* cutaneous incision in executing tenotomy; he gives to the blade of his bistoury a remarkable degree of narrowness and delicacy; he also takes the precaution to avoid detaching and separating the cellular substance surrounding the tendon—as well as to preserve, as far as possible, the integrity of its cellular sheath, believing this to have an important part to perform in the process of reparation; he gives the least possible pain consequently; opens few vessels, thereby avoiding ecchymosis, a constant attendant upon the operation as before practiced, which again circumscribes inflammatory action in the progress of re-union within very narrow limits. Finally, flexion of the foot is commenced *immediately* after the operation by the application of appariel for this particular object, avoiding the excitation of pain or uneasiness in the seat of operation.

"In operating, the patient lies in the recumbent position upon the breast and abdomen, the feet and legs projecting over the side of a bed or table, the foot supported by an aid, who, by exerting some flexion upon it, renders the tendo achilles somewhat tense. Then with the point of a thumb lancet or common bistoury, an incision of very limited extent is made through the integuments upon the side of the tendon, and in a longitudinal direction opposite its greatest prominence. Through the incision thus made, a narrow, convex, probe pointed bistoury is introduced, with which a passage is easily made, by separating the cellular substance, without endangering a puncture of the skin on the opposite side, a section of the tendon is made as in the procedure of Stromeyer.

"After practicing the operation as described four times with the greatest success, M. B. employed in a fifth operation another procedure, which consisted in the use of a narrow, blunt pointed *concave* bistoury, introduced through the cutaneous incision made as before, the instrument is then carried between the skin and the *posterior* face of the tendon, the latter is then divided from behind forward, giving to the wound a longitudinal direction. M. B. calls his instrument a *tenotome*, which from the thinness of its blade leaves but a very slight trace of its passage. A straight, sharp pointed bistoury may be made equally to subserve the purpose in executing the entire operation as described.

"One of the members of this commission, who invited M. Bouvier to practice this operation in the service with which he is charged, testifies to his ability and the excellency of his procedure, by which in eighteen days he succeeded in giving free use to the member of a man who had been deprived of it for 25 years.

"Delpech and Stromeyer allowed a certain period to elapse, when they supposed that the substance which serves as the medium of union between the ends of the divided tendon, had acquired a certain degree of consistence before attempting to restore the normal position of the foot. M. Bouvier, as already indicated, commenced the restoration of the foot *immediately* after the tendon had been divided, by applying an appariel, which not only maintained the degree of flexion already attained, but tended to augment it—at the same time preventing the inward inclination of the foot.

"The predecessors of M. B. temporised, fearful that the divided tendon might not re-unite. This apprehension is without foundation, inasmuch as we have seen in the case reported by Thelenius, that despite the wound of the integuments, and the extensive wound resulting from the tendinous division, and the separation of its margins, it cicatrized rapidly, and the patient walked with considerable promptness. The same is true of the observation reported by Sartorius."

In further illustration and confirmation of the truth of this position, it is only necessary to study the cases already reported from M. Mollinelli where we have seen that extensive *sections* of the *sphacelated* tendon were removed, in a succession of cases, wherein the *lost* structure was completely restored, and likewise the muscular and tendinous function. From this we pass to a subject of no less moment to the practical orthopedist and, we may add, of interest to the speculative physiologist—we refer to the physiology or mechanism of re-union of divided and *retracted* tendons. This subject has not been over-looked or neglected by M. Bouvier, and his experiments, as far as I am informed, are the only *conclusive* essays on the subject. We will give our readers an opportunity of forming their own opinions, by adducing the following important extract.

"The mechanism of re-union has been also studied by M. Bouvier. On the second or third day after tendinous section had been made, he found the cellular theca or sheath of the tendon thickened and more consistent than natural, and opened only the side where the instrument had penetrated, embracing both extremities of the divided tendon. Its internal surface was ecchymosed, of a bright red tint, in contact with itself or with the tendinous extremities which offered like discoloration of their surfaces.

"By the ninth day the connection which they formed was already consolidated, and adherent to the ends of a grey colored substance without any appearance of fibres. The contracted canal was without an opening, its walls being in contact and generally empty. M. B. has found it partly filled with partially coagulated blood.

It is toward the twelfth or thirteenth day that the canal begins to be effaced, and by the eighteenth it forms a resisting cord of like volume with the original tendon, and adhering to its extremities. The canal has nearly disappeared by this date, the tissue being compact and slightly infiltrated with a serous fluid begins to offer a fibrous structure.

"The twenty-fourth day the intermediate substance is found analogous to fibrous tissue, smaller than the tendon itself, endowed with great strength of resistance and solidity, no traces left of the inflammatory action which produced it.

"Examined the thirty-fifth day this substance was found perfectly continuous with the tendon but distinguishable from it.

"About the seventy-sixth day it presented the same appearances as the tendon of *another animal*, but distinguishable from it, by possessing greater solidity.

"With these facts, M. B. considers it demonstrated, that the formation of *new* tendon is due to the surrounding cellular tissue, first converted into a canal with distinct contiguous walls, which undergoes a gradual transformation into a solid fibrous cord.

The causes which concur to this re-union are, on the one hand, the natural adherence of the cellular tissue, from which the intermediate substance proceeds, with the external surface of the tendon—on the other hand the accidental adhesions of these fibres, with the ends of the divided tendon and the continuity which is thus established between these, and the tendinous fibres when they have *acquired* the *nature* of the latter."

The novelty of these conclusions respecting the mode of tendinous re-fomation cannot fail to engage the attention of the reader. If they should be established by subsequent experiments, they are decidedly subversive of the generally received doctrines of re-production of lost parts as applied to other organized animal structures. Nor can we subscribe hastily to this anti-Hunterian philosophy as applied to tendinous tissues without much limitation, especially as the facts of his experiments as detailed do not lead us to the same conclusions with our respected and learned author.

A case of some interest occurred several years ago in the Philadelphia Alms-house Infirmary, in the service of our learned and esteemed friend and late preceptor, Professor W. E. Horner, of the University of Pennsylvania, which reflects some additional light upon the subject of tendinous re-formation, and which may not be known to the profession. It will be found reported by Dr. H. in the Medical Recorder, or one of the early numbers of the "American Journal of the Medical Sciences."

The facts are briefly these—A case of rupture of the tendo achilles presented, and was treated after the ordinary surgery in such cases, viz.; by the employment of an appariel for maintaining extreme extension of the foot upon the leg. After waiting greatly beyond the usual period for solid *union* to take place, the ruptured tendon was found un-united, agreeably to the commonly received hypothethis on the subject. Under this embarrassing and apparently hopeless condition of the patient, Dr. H., doubtless influenced by the principles and practice first proposed and employed successfully by his venerable predecessor, the late Prof. Physick, in ununited osseous fracture, passed a seton between the ends of the retracted tendon. Continuing to act upon the same principles as in treatment of pseudo-arthrosis referred to, the seton was allowed to remain, until it was supposed that the requisite degree of inflammation was superinduced when it was withdrawn. After the lapse of some weeks, not exceeding two months, according to our recollection, the Doctor by preserving rest, extension of the foot, &c. of the limb had the happiness of witnessing the success of his operation, and the final restoration of his unfortunate patient. Query.—Was the cure in this case the result of mediate or immediate union of the ruptured tendon? We confess for ourselves, that, enlightened by the facts recorded by M. Mollinelli, as well as the circumstances which it seems to us must obtain in every case of divided tendo achilles, viz.; retraction and more or less *separation* of its extremities—we are decidedly inclined to the opinion already intimated, that original tendinous re-formation must take place in the process of reparation. It is also entirely inferable analogically, in the absence of closely observed and *noted facts* in reference to a decision of this important subject, that in no case of rupture of the tendo achilles is it possible to bring the ends of the retracted tendon into actual contact, and that the union must of necessity be *mediate*. It was this impression, doubtless, which induced Heister, De la Faye, and others of former times, to advise a suture of the tendon when divided, and it is even doubtful whether it could be accomplished *completely*, by as gross surgery as this would now be regarded. In every case of section of the tendon, which I have executed, or seen performed, a *chasm* is *immediately* seen and felt between the ends, which nothing but *re-deposition* can efface completely. A fatiguing and painful extension of the foot, continued for weeks and months, is not called for, then, as is the common practice in rupture of the tendo achilles—and enlightened by the facts of science, as they now stand recorded, this practice must and will be modified.

To return. It is but justice to my friend, Prof. Horner, to mention that, in a recent conversation with him in Louisville respecting his case of seton of the tendo-achilles, he considers it to be one of the *incipient* steps, which led to the adoption of an operation which it is our present object to consider. Whether, after the history of the subject already given, my readers will be induced to adopt a similar conclusion or not, the case is alike honorable to him who must be regarded, as he has been entitled, the father of American surgery, and to the gentleman immediately concerned.

There yet remains another subject to be mentioned, which may be considered as bearing some relation to that under consideration; my allusion is to *myotomy*, or section of the muscles. Apt illustrations of this operation will be found in that for torti-collis or wry-neck, by a section of the sterno-mastoid muscle; sections of the levator palpebræ superioris, of the sphincter ani; to which may be added the practice now employed with some success in Europe, in the treatment of certain deviations of the spinal column, by sections of one or more of the long muscles of the back; and, we may add, divisions of the contracted palmar aponeurosis. But it seems to me that tenotomy, for the cure of club-foot, could not have been deduced from any of the operations practiced upon the muscles, without original facts and experiments connected with the large tendons themselves. It would have been, in other words, reprehensible to attempt the cure of club-foot by a division of the tendo-achilles, without the most *indubitable evidence* that its function would not thereby have been destroyed, or even materially impaired; as such a result would have left the individual in an incomparably worse condition, than the most frightful deformity, with the capacity of tolerable locomotion. Again, original observations or experiments were indispensable, besides those referred to, because it is familiar to surgeons and pathologists, that muscular substance when lost, is not repaired by the re-deposition of the same tissue; and that its function consequently must be partially, if not completely lost.

Of this fact I was skeptical until a few years since, whilst a resident and practitioner of the city of Lexington, when the following case presented.

I was summoned in haste, a short distance into the country, to see a man who was bleeding from an incised wound of the leg, inflicted accidently by a scythe in mowing. Before my arrival, he had been transferred from the meadow to the house. The plexus of flexor muscles which lie against the outer aspect of the tibia and between it and the fibula had been deeply divided by a transverse wound, near the middle of the leg. The hæmorrhage from some divided branches of the anterior tibial artery, although profuse at first, was of short duration and did not require a ligature. The flexor communis was apparently divided, whilst the tibialis anticus and flexor proprius were not entirely severed. The ends of the divided muscles had, moreover, retracted, leaving a sulcus which admitted the ends of two or three fingers. A roller was applied methodically from the toes upward, and another from above the knee joint downwards, meeting at the wound with the design of approximating the retracted ends of the divided muscles as near as possible. As a farther means of fulfilling the same indication, the foot was maintained in extreme flexion. In a few days I learned that my patient had disregarded the injunctions of quietude, and had been upon his feet attempting to walk. The appareil was re-adjusted immediately, and several times afterwards necessarily, before the wound cicatrized, which took place in about 20 days. It is proper to state that it was impracticable by the most careful application of the rollers, &c. to completely obliterate the vacuity at the point of incision.

Some weeks after the wound healed I was informed that much suffering was experienced in every attempt to walk, and that more or less pain in the foot and lower part of the leg, was experienced at all times in the injured limb—with a request to visit him. Nothing remarkable at this time presenting in the appearance of the injured extremity, I was induced to attribute the remaining disability to a partial division of that plexus of the anterior tibial nerve found winding over the flexor communis, to be distributed upon the dorsum of the foot—and to conclude that his distress was neuralgic. No permanent benefit followed a prescription founded on this view of the case, and on examination it was found that a depression or gap existed in the original site of the injury, imparting through the skin a soft puffy sensation, and of sufficient magnitude to receive a finger, pressed longitudinally into it; the un-united condition of the divided muscular fibres was evident. Moreover, on every renewal of flexion of the foot, the injured muscles were found greatly at fault.

Early the ensuing autumn, several months after the accident, the disability continuing, the man was brought to town and submitted to the following operation. A longitudinal incision was made over the centre of the depressible point, and the retracted ends of the muscles exposed. The vacuity beneath the integuments was *partially* filled with *cellular substance* of different density—mostly lax and soft. This constituted the only medium of connexion between the retracted muscular fibres, which were variously separated from half an inch to an inch. I then removed carefully this cellular deposit denuding the ends of the muscles. By the aid of adhesives, the roller, posture, &c. the ends of the muscles were approximated as much as possible—but notwithstanding the greater faithfulness of my patient, a very manifest depression was still perceptible, weeks after the wound had closed.

Some improvement in the function of the member resulted from this operation, but disability continued for months afterward and as long as I had any knowledge of the case.

We will recur once more to the report upon M. Bouvier's memoir, for the purpose of epitomizing the four cases which he had then treated, as well as to present his general deductions upon the entire subject. All these cases were accidental, or acquired. Three of them were treated by M. B.; the 4th by M. Roux.

"The subject of the first was a young woman, 14 years of age, who as a consequence of scrofulous abscesses, with numerous cicatrices situated upon the posterior part of the leg, had a very aggravated club-foot, of the third variety, (equina.) The operation performed was after the manual of Stromeyer, slightly modified by Bouvier, and in 24 days the foot was restored to a right angle with the leg. The cure was perfect in three and half months notwithstanding the patient was not entirely governable.

"The second case was that of a man, aged 46 years, who had an accidental club-foot, of the same species as the preceding, produced by the bite of a dog on the heel, at his sixth year. The section of the tendon was practiced the 12th of

February. In twenty-five days the foot was restored to a right angle with the leg, and notwithstanding the formation of a cicatrix upon the heel, the result of improper application of the appareil—the fortieth day he walked with facility. The accidental ulceration required repose for a month.

"The third operation was performed upon a young woman, who had been the subject of hemiplegia of the right side for four years. The abdominal member remained feeble and the foot in permanent extension, which was forced outward and flexed. The operation in this case was practiced by M. Bouvier's second manual, i. e. from the cutaneous aspect of the tendon. In eight days the foot was flexed beyond a right angle, and in a short time the foot could be applied with the sole in a natural position upon the floor and without deviation—but the weakness of the muscles rendered progression unsteady.

"The fourth case belonged to M. Roux. This skilful operator practiced a section of the tendo achilles upon a boy of twelve years, who had been the subject of the same variety of club-foot, since his second year. The 4th of August, 1836, the operation was performed, and the foot brought immediately to near a right angle. The twenty-first day, re-union was complete, from which time progression was easy. This operation was also executed after the second manual of Bouvier.

"The section of the tendo achilles is manifestly an improvement in the treatment of club-foot. The length of time occupied in its treatment by machinery, and the painfulness of the process will not admit of comparison with the brevity of treatment, &c. by a section of the tendo achilles."

M. Bouvier concludes his memoir with the five following propositions, which we consider just.

"1st. Section of the tendo-achilles promptly cures phalangeal club-foot at an age when machinery could exert but limited influence, and in cases hitherto reputed incurable.

"2d. The operation (after my own method,) is as easy in its execution, as it is safe in its results.

"3d. Cures thus obtained can be regarded as permanent, since the patient treated by Delpech twenty years before, enjoyed the complete success of the operation.

"4th. The mechanism of re-union conclusively demonstrates, that the *immediate* separation of the ends of the divided tendon is no obstacle to the re-production of a new tendon equally capable as the original one of supporting the ordinary efforts of the muscles.

"5th. Section of the tendo-achilles abridges the cure of internal club-foot, when accompanied by a forcible retraction of the extensors of the foot, and allows us to expect a more perfect restoration than has been heretofore obtained by machinery alone."

From my earliest study of practical tenotomy in 1837, '38, during a professional visit to Europe, I became convinced, that if the principles upon which it is predicated, were correct, as I believed them to be, its application would not be confined to those tendons concerned in deformities of the foot alone, and determined to embrace the earliest opportunity for testing the correctness of this opinion. I am now happy to say that I have had no reason to modify this opinion, then formed, in any of its applications (some of which are novel,) which I have had an opportunity of making; and that I do now regard tenotomy as established upon fundamental, scientific principles, susceptible of application wherever permanent muscular and tendinous contractions are concerned in the production and maintenance of deformities.

What remains of this essay, will be appropriated to reports and remarks upon some of the cases of deformity in which I have been consulted, with the results of treatment by a section of the tendons, &c.

*Case* 1*st*. The first in order of treatment was that of the Rev. Dan'l S. Colgan, of Columbia, Ky.

As a consequence of an extensive burn of the fore-arm, hand, and fingers of one extremity, from accidentally falling into the fire in the absence of his nurse when a child of 18 months old, the fore finger was lost to within about two lines of its first phalangeal articulation, and the middle, ring, and little fingers permanently contracted to various extents. The middle finger was rather more than half flexed upon the hand, resembling a hook; the ring finger was even more flexed, chiefly bent at its first phalangeal articulation, whilst its two last or distal phalanges were applied nearly flat upon the palmar aspect of the hand, between which and the fingers cutaneous connections existed to within two or three lines of its extremity. The little finger was so much distorted as to have suffered partial dislocation at all its articulations, and in the process of cicatrization so completely buried beneath the integuments of the hand as to elude observation, without a careful inspection. It was thought advisable not to interfere with it, particularly as it presented no actual deformity. The middle finger was first operated upon, the 11th February, 1839, in the presence of, and aided by, professional and other friends of Louisville.

A longitudinal incision of the integuments was first made to the extent of six or eight lines upon the palmar aspect of the finger between its first and second articulations. A small opening was then made through the theca of the flexor tendons, through which the delicate *tenotome* of Bouvier, already described, was introduced, and the sublimis and profundus tendons transversely divided. By very gradual extension, the finger was brought immediately to form a line with the hand, and was thus maintained by a roller and splint applied upon its dorsal surface. The margins of the external incision were approximated by adhesive strips and simple dressings applied. It is worthy of remark that partial *ligamentous* anchylosis at the first metacarpal joint offered considerable temporary resistance in extending the finger, after the principal cause was overcome.

By the 19th of the same month, the wound being nearly cicatrized, and the improved posture of the finger giving Mr. C. satisfaction, at his solicitation the ring finger was subjected to a somewhat similar operation and treatment. The extensive cutaneous adhesions described, first required division, together with the palmar aponeurosis appropriated to this finger. Tendinous section was then executed as in the first operation, and the finger extended and dressed in a similar manner. From the wide gap in the integuments, which necessarily presented when this finger was restored, cicatrization was not complete before the 22d of March, on which day the gentleman left for the country. At this time both members, subjected to treatment, were nearly in a line with the hand, which posture he was advised to preserve for some succeeding weeks.

It was particularly observed during the successive divisions of the skin, aponeurosis, and tendons, executed in the last operation, that the obstacle to extension was not overcome until the section of the tendons was completed.

From the nature and extent of the injury, thirty-five years before, producing deep and extensive cicatrices of the fore-arm, hand, and fingers, with *anchylosis* of the first phalangeal articulations, more than partial recovery of the *functions* of the fingers was not to be expected, nor was promised. Some months succeeding the treatment, however, I had an opportunity of inspecting them, when some power of flexion and extension was enjoyed—the middle finger could be brought in apposition with the thumb and thus made to subserve many valuable purposes. In addition to which Mr. C. was greatly gratified with the result, as he could now present his *fingers* with the hand and arm in the extended position, so essential in gesticulation.

*Case 2d*, Was that of a colored girl, Cornelia, aged about 10 years, belonging to Mrs. J., of this city. She was represented as being of healthy conformation at birth. This was preserved until she was about thirty months old, walking with the ordinary facility of children of that age, when she was attacked with pains in her lower extremities. At the expiration of about three months these *pains* subsided, leaving *both* lower extremities extensively deformed, in such manner and degree that she has not been able to move in the erect posture since. Her general health has been good. This is the brief and imperfect history of the case obtained up to the month of April, 1839, when she was first submitted to particular examination.

The deformities were found to involve five principal articulations, i. e. she had double *Kyllopodia Equina,* inclining to *K. vara,* (the feet and toes tending inward,) both legs flexed upon the thighs to less than a right angle, and from a slight contraction of the tensor vagina femoris of the left side, the thigh was slightly flexed upon the pelvis.

By reference to the cut, (Fig. 1.) taken from a drawing which I made from a cast of the deformity of the *left* extremity, prior to the operation, a correct estimate may be formed of the *double* deformity of the foot, and at the knee joint, and of *both* limbs, with a very slight exception. The difference between the limbs was to be observed in the greater atrophy of the left, from which the cast was taken, in addltion to which the flexion of the leg was greatest in this limb, whilst the extension and curvation of the right foot was greater than the left. The resemblance of the deformities, however, in kind and degree in comparing the limbs was so great as to supercede the necessity of delineating both.

The trunk and upper extremities were developed greatly beyond one of her years—which doubtless is referable to the extraordinary exercise to which they were subjected in her method of progression in the *sitting* position. The arms were employed in moving, not unlike *crutches,* by successively placing them in front of the body, resting upon the palms of the hands, and drawing the body after them. The weight of the trunk, &c. rested principally upon the *right* nates, thigh, and leg.

After this manner, the feet and legs drawn close beneath, and to the side of the *left* nates, she was enabled to perform a species of locomotion upon a plane surface, and to ascend and descend, at a slow progress, the stair way. After what has been stated, it will readily be perceived why the *left* limb generally was less developed than the right.

The 19th day of April, 1839, I performed tenotomy upon the right tendo achilles. I had on this occasion the presence and kind assistance of several professional friends of Louisville. The foot was easily flexed to some extent immediately after the operation, so that the deformity was reduced about one-half, the ends of the divided tendon were found separated six to eight lines, and by the fourteenth day the foot had been gradually brought, by the employment of an appareil devised for the case, to a right angle with the leg.

The slight incision of the integuments, but little greater than that of common venesection, adhered in twenty-four hours, and by the twenty-first day the chasm between the ends of the divided and retracted tendon was completely filled, leaving no sensible inequality.

The 11th of May just three weeks after the first operation, I executed a section of the tendon of the biceps flexor cruris of the same (right) limb.

By the seventh day after this operation the leg had been gradually extended to a straight line with the thigh, by an apparatus somewhat similar to that of Amesbury's for fractures. This appareil, devised for the occasion, maintained the foot simultaneousiy in a normal position with the leg. In about a month this was removed and some motion commenced at the knee and ankle joints. The capacity for complete flexion and extension of the leg upon the thigh was rapidly acquired, and appeared to be perfect in degree and force, after a month to six weeks daily exercise. During the application of the last apparatus, it was subsequently ascertained, the patient had from time to time clandestinely removed the foot from the shoe attached to the heel piece, by which the heel rested against the os calcis.

This is mentioned as a circumstance which should be scrupulously guarded against under like circumstances, inasmuch as soreness and ulceration are the almost certain consequences. This unpleasant effect resulted in the present instance, creating a delay of many weeks in the progress of the case.

In the months of July and August succeeding, the first exercise in the *erect* posture was commenced, by the aid of crutches. These essays were regarded as sufficiently flattering, notwithstanding the want of muscular power and remaining soreness complained of in the recently *ulcerated* surface. Exercise of the limb was now resumed in the *sitting posture*, and maintained daily, for several months with as much assiduity as could be attained, with a view of developing size, strength, &c. In this I was not disappointed, as its increase in bulk and strength was very noticeable, and by the month of November the patient again commenced exercise erect with less timidity and fatigue than before. Notwithstanding, a few minutes in the erect posture excited a great degree of muscular tremor and uneasiness in the limb generally.

The *contrast* in the size of the extremities increasing, the right continuing to augment in *length* as well as bulk, determined me to treat the left *immediately*, if for no other purpose than to favour its growth—its *prospective* aid in erect locomotion being all that was *then* anticipated.

The 3d December I made a section of the biceps flexor cruris on the one hand, and of the semi-tendinosus and membranosus on the other, at my private lecture room, aided by several professional friends, and in the presence of a number of medical students and other gentlemen.

In about twelve days from the date of this operation, the leg had been gradually extended in a line with the thigh, and thus maintained about two months before muscular motion was allowed. Circumstances progressing favourably, tenotomy upon the *left* tendo-achilles was performed the 20th January 1840. The eighteenth day succeeding this operation, by gradual movements of the apparatus every second day, the foot was brought slightly *within* a right angle. The modified appareil of Amesbury, with the foot piece, was now applied as before, fulfilling like indications. This was worn about two months, and tendinous re-formation being considered perfect, it was permanently withdrawn and the freest muscular motion allowed and encouraged. Attention was now particularly directed to the *right* extremity again, the main dependence for immediate *erect* locomotion.

It had continued to develope in the interval of treatment bestowed upon the left, and extension and flexion of the leg were considered *perfect* by several professional gentlemen who saw and examined it with me; notwithstanding, *soreness* and *pain* were complained of in the *tendo-achilles*, whenever she walked.

The unfortunate ulceration of the integuments covering the heel, had in fact caused some *contraction* of the tendon, and consequent extension of the foot *beyond* a *right* angle upon the leg.—This disability not improving by exercise up to the early part of April last, determined me to operate a *second* time upon the right tendo-achilles.

A *re-section* of this tendon was accordingly executed on the sixth day of April, and an apparatus immediately applied by which in a few days the foot was brought into *extreme* flexion upon the leg. Considering the *second* tendinous union as solid by the 1st of June, the patient commenced to walk again with her crutches, under very *improved* circumstances. By frequently repeated, daily exercise she was soon capacitated to walk about the house and yard, in considerable comfort, with increasing strength and assurance.

About the middle of the present month, (August,) I find my patient continues to improve in every respect, so much so, that she is capable of walking or standing upon her feet two hours or more uninterruptedly with but little fatigue. In addition, the left limb within the last few days has been brought into requisition, and aids materially in standing or progression, by resting upon the points of the toes—the heel of this limb not reaching the ground by about two inches and a half.

Her general health remains, as it has been, uniformly good. No inequalities in the the tendon operated upon exist, nor is there any perceptible want of the most perfect continuity and consolidation of structure at the points where tendinous sections were made. Of this I am fully convinced from a recent examination made in connection with my friend, Prof. Horner, who equally satisfied himself on this subject.

The delineation marked No. 2, of the accompanying cut, is from a drawing from nature which I made of one of the restored extremities, about two weeks since—it represents the present position of it, in an erect or standing attitude.

Remarks.—The multiplicity of deformities, complicating this case, and destroying erect locomotion for a period of nearly nine years—taken in connection with the success attending the application of the principles of tenotomy to the tendons of the flexor *muscles of the legs*, renders it, so far as my observation and reading extend, *entirely unique*. It is, besides, well calculated to encourage us in subsequent applications of tenotomy in similar pathological conditions, in *whatever part located*. This leads me to allude to a highly important application of the same principle to the treatment of strabismus or squinting, by Prof. Dieffenbach, of Germany. This ingenious surgeon has divided the internal rectus muscle (or rather, tendon I conclude,) of the eye in internal strabismus, with success. Several similar cases have appeared in the "London Lancet,"* in which this operation has been successful in the hands of P. B. Lucas, Esq.

And we conclude that the same operation may be applied to the rectus externus, internal oblique, and tendons of other muscles of the eye, where these are found concerned in the production of abnormal direction of the axis of that organ.

The same pathological states will, no doubt, be found in the muscles and tendons of the thoracic extremity, besides the illustrations contained in the first cases which I treated.

This essay might be protracted, by considering certain subjects *indirectly* connected with tenotomy, but it has already equalled the limits contemplated in the outset.

Reference is more particularly had to the manuals, which I have found safest for executing tenotomy at the popliteal region—together with Wm. Bennet's details of his modications of Dieffenbach's operation upon the rectus internus oculi.

The most suitable age for treating congenital Kyllopodia, when this can be selected, as well as the whole subject of anchylosis, the correct diagnosis of which becomes so indispensa-

---

*London Lancet, May 2d, 1840. p. 188.

ble in the interpretation of deformity in certain cases, are not less intimately associated with our subject.

I have found it safest in operating at the ham to make the preliminary incision through the skin on the *inner side* of the centre of the flexor tendons, and to introduce the tenotome from *within outward*, so as to make the section of the tendons from *without inward*. This is founded upon the relations sustained between the popliteal nerves and the tendons. These nerves after descending to about the centre of the popliteal space, are found in the remainder of their course through this region, to lie almost in *contact* with the *inner aspect* of the flexor tendons. The section of the tendon being made from without inward, so soon as the section is complete, (evidenced by its sensible contraction,) the instrument should be withdrawn and the nerve is safe, whereas it is possible if passed first around the inner side of the tendon the accompanying nerve will be embraced.

This modification I have also made in operating upon the tendo-achilles with a similar view of avoiding a wound of the posterior tibial artery and nerve.

In only one instance have I declined operating for Kyllopodia from the tender *age* of the patient.

This was an interesting and vigorous child of *four months old*, brought by its parents from a distant state last year, which I advised to be brought back when it had attained its second year.

Doubtless tenotomy, so far as this alone is concerned, might be safely executed at a very early age, and when the torsion of the foot would offer the least obstacle to redress, but I should not feel inclined to impose the necessary restraints of the restoring appareil when the excitability of the nervous and vascular systems are known to be most exalted. From the fifth to the eighteenth year, must be considered as a very favorable age for treating any of the varieties of Kyllopodia. In illustration of the third and last topic named, of collateral importance in *practical tenotomy*, I will only add the following instructive case, in which tenotomy was *unsuccessful.*

Mrs. M. at about forty years, as a consequence of acute articular rheumatism, three years before, of the right knee joint, had the leg permanently flexed upon the thigh, about one third—i. e. it formed a very open angle with the thigh. The leg could be *permanently* flexed, but did not admit of extension beyond the degree named, and consequently lameness existed. It was evident enough that partial anchylosis existed in the knee joint, but inasmuch as the flexor tendons of the leg were rendered tense, in efforts at complete extension of the leg, the degree of agency which these exerted in maintaining the abnormal posture could not *a priori* be decided. Besides, preliminary diagnostic essays were resorted to with the original apparatus of Amesbury for fractures, with *perceptible extension* of the leg, beyond what she could *voluntarily* accomplish.

Encouraged somewhat by these trials, and knowing the doubtful nature of the result, this lady solicited the operation of tenotomy.

This operation was accordingly executed about the middle of May, 1839, in which sections of the biceps flexor cruris, and of the semi-membranosus and semi-tendinosus were executed. The limited cutaneous incisions were brought together by adhesives and united without suppuration. The third day succeeding these operations the appareil named was re-applied and by slight and repeated movements in the rack of the machine, the limb was extended *beyond* what it had been in the preliminary treatment.

Nothing was gained after this time, as further effort at extension of the limb produced pain and soreness in the *joint*, and after a short time, all further treatment was abandoned.

Tendinous reformation was as speedy and perfect in this, as in the preceeding operations upon the tendons of the flexors of the leg, and in due time the power of flexing the limb was recovered.

From all the facts as well as the history of Tenotomy in our possession, we feel authorized in advancing the following conclusion, viz.; that there is no variety of Kyllopodia, or any of its *pathological congeners*, recent or old, congenital or acquired—uncomplicated with anchylosis—which is not remediable in a very large proportion of instances by the treatment which we have endeavored to elucidate, and that the most aggravated of these deformities, from age or otherwise, may in like manner be decidedly ameliorated.

*August* 24*th*, 1840.

## Figures and Tables

**Fig. 1 f1:**
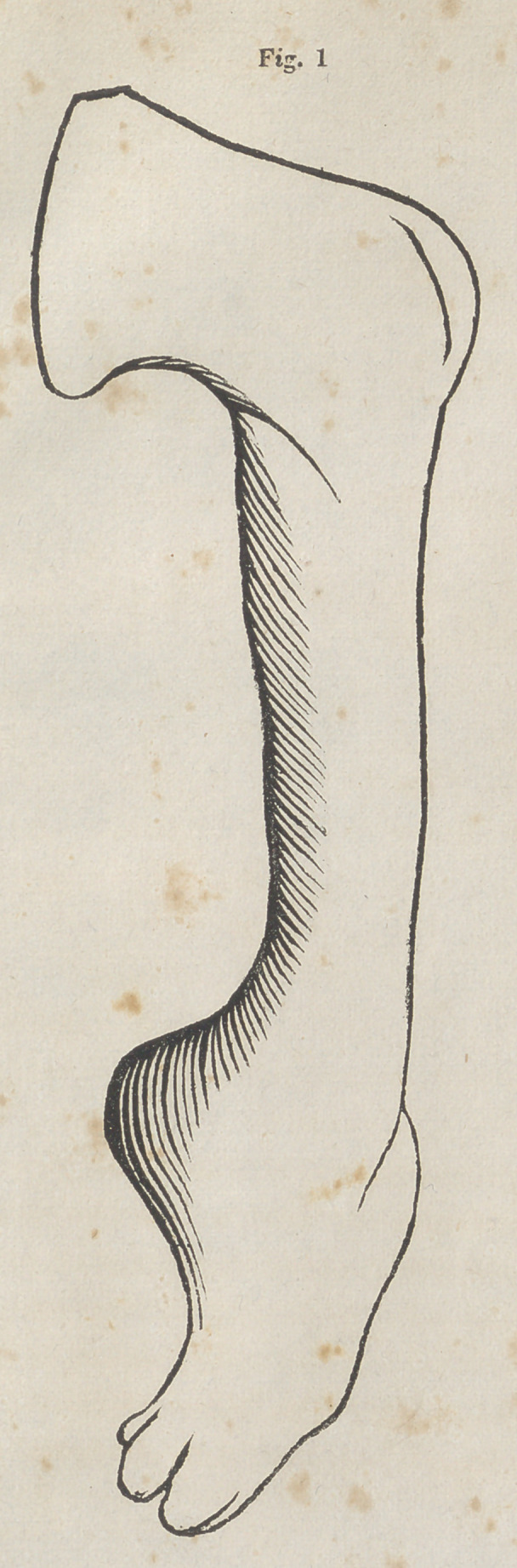


**Fig. 2. f2:**