# Tenotomy—a Correction

**Published:** 1840-10

**Authors:** 


### TENOTOMY—A CORRECTION.

To insure a correct and well executed wood cut to accompany the Essay on Tenotomy, the publishers of this Journal sent the original drawings from nature, furnished by the author, to a Cincinnati artist—we are sorry that we are unable to publish his name. Besides the coarseness of the cut, which must strike every eye, its want of accuracy is such as to demand a brief explanation, in order that an erroneous impression of the success of Dr. Richardson's operation be not conveyed. The *ensemble* of the extremities, before as well as after treatment, is much more heavy and clumsy than the drawings indicated; in addition to which, the measurements around the heel and instep of the deformed member are nearly onethird too great as they are represented in the plate. The same want of observance of the drawings exists in the representation of the restored member, in which the instep is so much elevated as to resemble a shoemaker's last, and the foot appears too much extended, whilst the leg, which in the original drawing was ***completely*** extended on the thigh, appears in the wood cut partially flexed. Y.

